# Effects of novel glucose-lowering drugs on the lipid parameters: A systematic review and meta-analysis

**DOI:** 10.1016/j.amsu.2022.103633

**Published:** 2022-04-16

**Authors:** Sophia Dar, Ahmed Kamal Siddiqi, Tamim Omar Alabduladhem, Ahmed Mustafa Rashid, Saba Sarfraz, Talha Maniya, Ritesh G. Menezes, Talal Almas

**Affiliations:** a Hackensack University Medical Center, Hackensack, NJ, United States; b Department of Medicine, Ziauddin Medical University, Karachi, Pakistan; c College of Medicine, Imam Abdulrahman Bin Faisal University, Dammam, Saudi Arabia; d Department of Medicine, Jinnah Sindh Medical University, Karachi, Pakistan; e Department of Medicine, Islamabad Medical and Dental College, Islamabad, Pakistan; f Department of Medicine, Royal College of Surgeons in Ireland, Ireland

**Keywords:** SGLT2 inhibitors, GLP-1 agonist, DPP-4 inhibitors, Meta-analysis, Lipid parameters

## Abstract

**Aim:**

We aim to evaluate the impacts of sodium-glucose co-transporter 2 inhibitors (SGLT-2), glucagon-like peptide 1 agonist (GLP-1RAs), and dipeptidyl peptidase-four (DPP4) inhibitors on the levels of high-density lipoprotein, low-density lipoprotein, triglyceride and total cholesterol.

**Methods:**

The MEDLINE database was searched from inception till October 2021, for randomized controlled trials assessing the effects of sodium-glucose co-transporter two inhibitors (SGLT-2), glucagon-like peptide 1 agonist (GLP-1RAs), and dipeptidyl peptidase-four (DPP4) inhibitors on lipid levels.

**Results:**

A total of 57 trials were included in the analysis. Our pooled analysis demonstrates that SGLT-2 inhibitors significantly increase the levels of HDL (WMD = 0.07 mg/dL [0.06 to 0.08], P < 0.00001). SGLT-2 inhibitors were also found to be significantly associated with an increase in the levels of LDL (WMD = 0.11 mg/dL, [0.09–0.13 mg/dL, P < 0.00001). Pooled analysis also demonstrates that SGLT-2 inhibitors significantly reduce the levels of triglyceride (WMD = −0.10 mg/dL, [-0.13 to −0.06], P < 0.00001). Our pooled analysis demonstrates that SGLT-2 inhibitors significantly increased the levels of total cholesterol (WMD = 0.10 mg/dL, [0.06 to 0.15], P < 0.0001), whereas, GLP-1RAs significantly reduced the levels of total cholestrol (WMD = −0.18 mg/dL, [-0.34 to −0.02], P = 0.03).

**Conclusion:**

This is the first head-to-head study comparing the effects of 3-novel glucose-lowering agents to lipid parameters. However, more trials are crucial to better understand the impact of glucose-lowering drugs on lipid parameters.

## Highlights

• This systematic review and meta-analysis evaluated the effects of novel glucose lowering agents on the levels of high-density lipoprotein, low-density lipoprotein, triglyceride and total cholesterol.
• We found that SGLT2 inhibitors show a significant increase in LDL and HDL.
• More studies are required to assess the effects of DPP4 inhibitors.

## 1 Introduction

Nonalcoholic fatty liver disease (NAFLD) is the leading cause of chronic liver disease globally. It is estimated to affect 70–80% of people with type 2 diabetes mellitus (T2DM) [1,2]. NAFLD has lately arisen as a significant health hazard among Asia's obese population, with NAFLD prevalence in Asia being estimated to reach 29.6%, perhaps exceeding that in Western countries [3,4]. There is significant evidence of the coexistence of the two major comorbidities: NAFLD and T2DM. Evidence exists of an increased risk of developing nonalcoholic steatohepatitis (NASH), cirrhosis, hepatocellular carcinoma, cardiovascular complications, and many other consequences with chronic uncontrolled diabetes [[5], [6], [7]]. Thus, early identification and monitoring of NAFLD and NASH in persons with T2DM are critical. Despite the condition's importance, there is currently no recommended therapy for T2DM patients who have NAFLD.

The primary line of care is weight reduction by adopting a healthy lifestyle (i.e., changes in diet and exercise). Unfortunately, weight loss by lifestyle changes alone is often a challenge for obese patients [8]. According to a study, fewer than half of patients can reduce their weight with lifestyle interventions alone [9]. Based on equivalent pathophysiological pathways shared by T2DM and NAFLD, glucose-lowering drugs treating T2DM are effective in NAFLD patients, which can enhance glycemic control and have a beneficial effect on the liver [10]. The two incretin-based drugs, dipeptidyl peptidase-four inhibitor (DPP-4) and glucagon-like peptide-one (GLP-1RAs) agonists, along with sodium-glucose co-transporter (SGLT-2) inhibitors are novel classes of glucose-lowering drugs used in the management of T2DM.

It is plausible that GLP-1RAs may effectively treat NAFLD by increasing satiety and decreasing hunger while also delaying stomach emptying. DPP-4 inhibitors are designed to keep GLP-1RAs agonists from degrading, thereby extending their half-life [3]. T2DM and NAFLD patients benefit from the SGLT-2 inhibitor class because it inhibits the renal ability to reabsorb filtered glucose and so improves glycemic management, body weight, and blood pressure [11].

Multiple randomized controlled trials (RCTs) have demonstrated the potential benefits of GLP-1RAs, DPP-4 inhibitors, and SGLT-2 inhibitors for treating NAFLD in diabetic patients. They all have yielded positive results individually [11]. However, previous studies have analyzed the impact of SGLT-2 inhibitors, GLP-1RAs and DPP-4 inhibitors on hepatic enzymes and fibrosis but have failed to assess the effect on hepatic fat content. Moreover, no study yet evaluates the effectiveness of the three glucose-lowering drug classes in T2DM patients with or without NAFLD. Currently, no existing literature provides the evidence of the effectiveness of 3-novel glucose-lowering drug classes in NAFLD patients with or without T2DM and hence, there is no clear consensus for the management of such patients. Although, studies have been conducted previously, assessing the impact of the 3-novel glucose-lowering drugs on liver enzymes, unfortunately there is still a gap in knowledge and ambiguity regarding the efficacy and impact of the 3 drug classes on hepatic fat content in NAFLD patients with or without T2DM. In this light, we fill this gap in knowledge by conducting a head-to-head analysis and assessing the efficacy of the three-novel glucose-lowering drug classes (SGLT-2 inhibitors, GLP-1RAs and DPP-4 inhibitors) and their effectiveness on liver fat content i.e., HDL, LDL, triglycerides and total cholesterol in patients with T2DM with or without NAFLD as our primary outcome. As our secondary outcome, we have also conducted a subgroup analysis in patients with only T2DM to assess the impact of these three-novel glucose-lowering drug classes on hepatic fat content as mentioned above.

## 2 Methods

This systematic review was conducted and reported the following: Cochrane and PRISMA (Preferred reporting items for systematic review and Meta-analysis) 2020 guidelines [12]. The current study is noted to be well compliant with the AMSTAR 2 guidelines, with the quality of the present systematic review noted to be low in line with the guideline [13]. This study did not require an institutional board review approval since it includes data that is publicly available.

### 2.1 Search strategy and eligibility criteria

Two authors (AMR and AKS) independently conducted an extensive search on the MEDLINE database, from inception till October 2021, without any language and time restrictions, for randomized controlled trials reporting the outcomes of interest for any of the 3 novel glucose lowering drugs (Sodium-glucose co-transporter two inhibitors; Glucose-like peptide-1 receptor agonist; and Dipeptidyl peptidase-4 inhibitors), compared with placebo or other active glucose-lowering agents. To make certain that no important publication was missed, we used snowballing approach and conducted hand searches of all reference lists of eligible articles in order to avoid missing any relevant article. All retrieved articles were transferred to Endnote X7 (Clarivate Analytics, PA), to identify and remove duplicates. Finally, if studies from same author or institution was conducted in the same period and reported the same outcomes with a suspected overlap, only the latest study was included in this meta-analysis of the respective outcome. The included participants were patients with T2DM. The outcomes of interest were levels of high-density lipoprotein (HDL), low-density lipoproteins (LDL), triglycerides (TG), and total cholesterol (TC). All observational studies (cross-sectional, case-control, cohort, case reports) and animal studies were excluded. We mention the search string in supplementary data Table 1*.*

### 2.2 Data extraction and quality assessment

According to the inclusion criteria, two authors (AMR and AKS) independently short-listed trials based on titles and abstracts. Then the full texts of the articles were reviewed, and trials were included if they reported one or more outcomes. A third author (TJS) was consulted to solve any disagreements. For each included study, the following data was extracted: General information, study characteristics, interventions and outcomes (as mentioned earlier). As randomized controlled trials were included in this study, Cochrane risk of bias tool was used to assess the included trials’ quality [[14], [13]]. The following aspects were assessed: selection bias, performance bias, detection bias, attrition bias, reporting bias, and others.

### 2.3 Statistical analysis

Data analysis was performed using RevMan, Version 5.4 (Nordic Cochrane Center, Copenhagen, Denmark). The results were presented as weighted mean difference (WMD) with 95% confidence intervals (CIs) and were pooled using a random-effects model [15]. Results of the pooled analysis were visualized using forest plots. The Higgins Ι^2^ statistic was analyzed to assess heterogeneity. A value of less than 50% for Ι^2^ was considered acceptable. A p-value of less than or equal to 0.05 was considered significant in all cases. Importantly, the present work has been reported in line with the PRISMA guidelines [16]. Subgroup analysis was performed on RCTs reporting results on only patients with T2DM, without any comorbidities. Outcomes assessed in the subgroup analysis were HDL, LDL, TG and TC.

## 3 Results

### 3.1 Study selection, trial characteristics and quality assessment

**Fig. 1:**
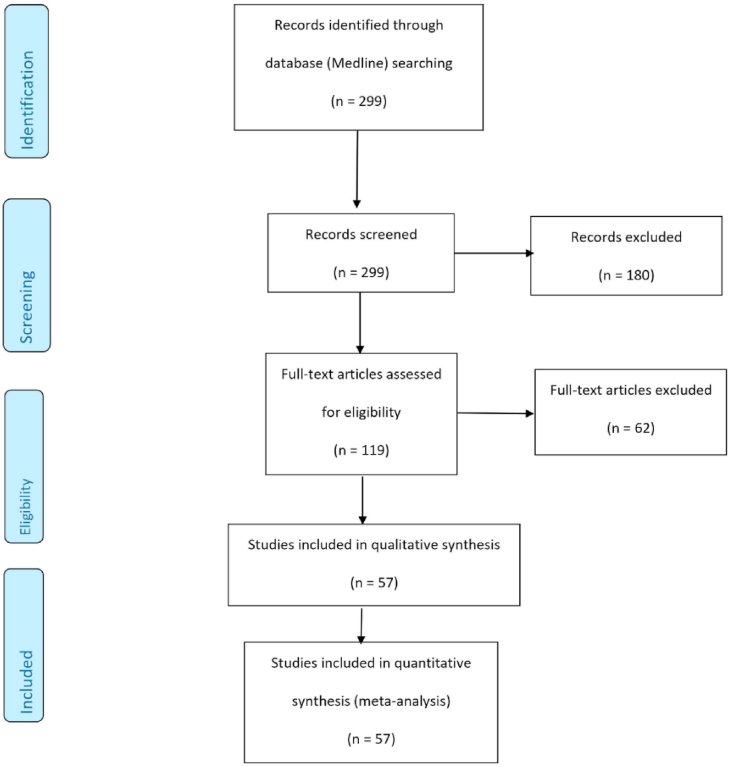
Summary of study selection process.

After a thorough search, 299 articles were identified. A total of 180 were excluded after reading their title and abstract. After a full text review, we excluded 62 articles. A total of 57 articles met our inclusion and exclusion criteria. PRISMA flowchart, summarizes the study selection process (Fig. 1). The characteristics of the trials included in our analysis are presented in Supplementary Table 2. The total number of participants was 18,742 and the range of study duration was between 12 weeks and 312 weeks. Among the 57 included studies, 35 studies reported results on SGLT-2 inhibitors, 11 studies reported results on GLP-1RAs, and 11 studies reported results on DPP-4 inhibitors.

Overall, the quality of the trials had low risk of bias. However, two trials [17,18] had a high risk of selection bias and failed to highlight random sequence generation. However, none of the included trials had a high risk with allocation concealment. Eight trials [[19], [20], [21], [22], [23], [24], [25], [26]] had performance bias due inadequate information regarding blinding of participants and personnel. Six trials had detection bias due to lack of blinding of outcome assessment [[20], [21], [22],^24^,^25^,^27^].

### 3.2 HDL

**Fig. 2:**
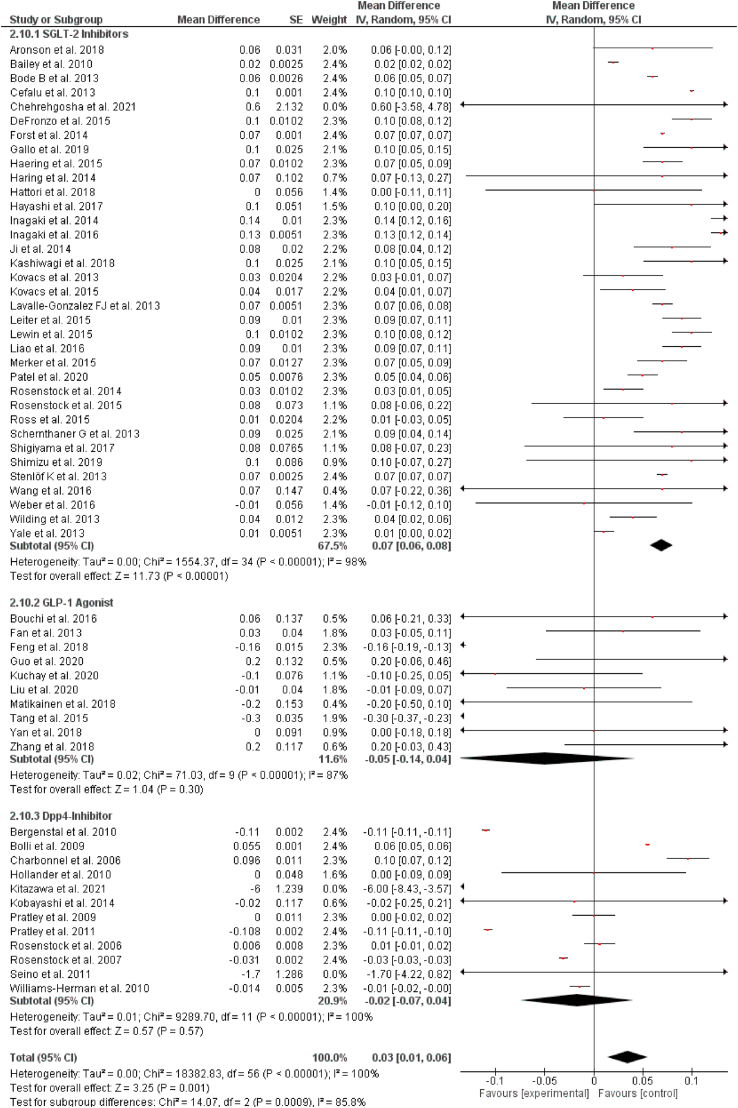
Pooled analysis of trials demonstrating effect of sodium glucose co-transport 2 inhibitors, glucagon-like peptide 1 agonist and dipeptidyl peptidase-4 inhibitors on HDL.

Out of 57 selected studies, 35 reported results for changes in HDL for SGLT-2 inhibitors. Our pooled analysis demonstrates that SGLT-2 inhibitors were significantly associated with increasing the levels of high-density lipoprotein (HDL) (WMD = 0.07 mg/dL, 95% CI: 0.06–0.08 mg/dL, P < 0.00001). Out of 57 selected studies, 10 reported results for changes in HDL for GLP-1RAs. Our results demonstrates that GLP-1RAs had no significant effect on the HDL levels (WMD = −0.05 mg/dL, 95% CI: −0.14 to 0.04 mg/dL, P = 0.30). Out of 57 selected studies, 12 reported results for changes in HDL for DPP-4 inhibitors. Similarly, DPP-4 inhibitors were also not significantly associated with changes in the levels of HDL (WMD = −0.02 mg/dL, 95% CI: −0.07 to 0.04 mg/dL, P = 0.57). The results are summarized in Fig. 2.

### 3.3 LDL

**Fig. 3:**
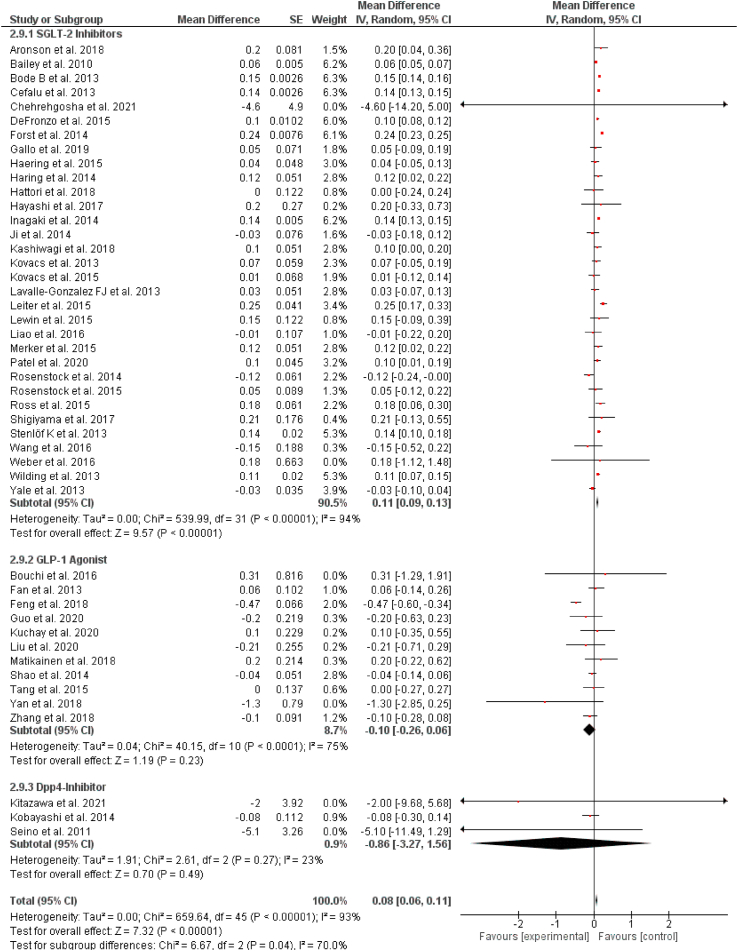
Pooled analysis of trials demonstrating effect of sodium glucose co-transport 2 inhibitors, glucagon-like peptide 1 agonist and dipeptidyl peptidase-4 inhibitors on LDL.

Out of 57 selected studies, 32 reported results for changes in LDL for SGLT-2 inhibitors. Our pooled analysis demonstrates that SGLT-2 inhibitors significantly increase low-density lipoprotein LDL levels (WMD = 0.11 mg/dL, 95% CI: 0.09–0.13 mg/dL, P < 0.00001). Out of 57 selected studies, 11 reported results for changes in LDL for GLP-1RAs. Our results demonstrate that GLP-1RAs had no significant effect on LDL levels (WMD = −0.10 mg/dL, 95% CI: −0.26 to 0.06 mg/dL, P = 0.23). Out of 57 selected studies, 3 reported the effects of DPP-4 inhibitors on LDL levels. Our results demonstrate that DPP-4 inhibitors were also not significantly associated with changes in LDL levels (WMD = −0.86 mg/dL, 95% CI: −3.27 to 1.56 mg/dL, P = 0.49). The results are summarized in Fig. 3.

### 3.4 Triglycerides

**Fig. 4:**
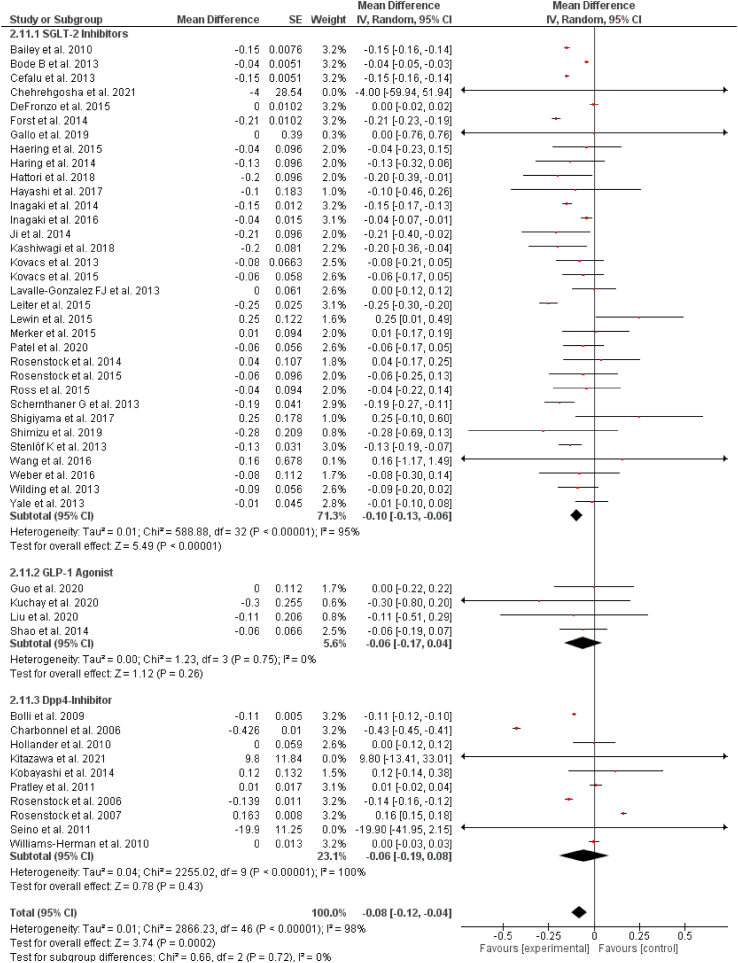
Pooled analysis of trials demonstrating effect of sodium glucose co-transport 2 inhibitors, glucagon-like peptide 1 agonist and dipeptidyl peptidase-4 inhibitors on triglycerides.

Out of 57 selected studies, 33 reported the effects of SGLT-2 inhibitors on triglycerides (TG) levels. Our pooled analysis demonstrates that SGLT-2 inhibitors significantly reduce the levels of TG (WMD = −0.10 mg/dL, 95% CI: −0.13 to −0.06 mg/dL, P < 0.00001). Out of 57 selected studies, 4 reported results for changes in TG levels for GLP-1RAs. We found that GLP-1RAs had no significant effect on TG levels (WMD = −0.06 mg/dL, 95% CI: −0.17 to 0.04 mg/dL, P = 0.26). Out of 57 selected studies, 10 reported results for changes in TG levels for DPP-4 inhibitors. Our results also demonstrate that DPP-4 inhibitors had no significant effect on TG levels (WMD = −0.06 mg/dL, 95% CI: −0.19 to 0.08 mg/dL, P = 0.43). The results are summarized in Fig. 4.

### 3.5 Total cholesterol

**Fig. 5:**
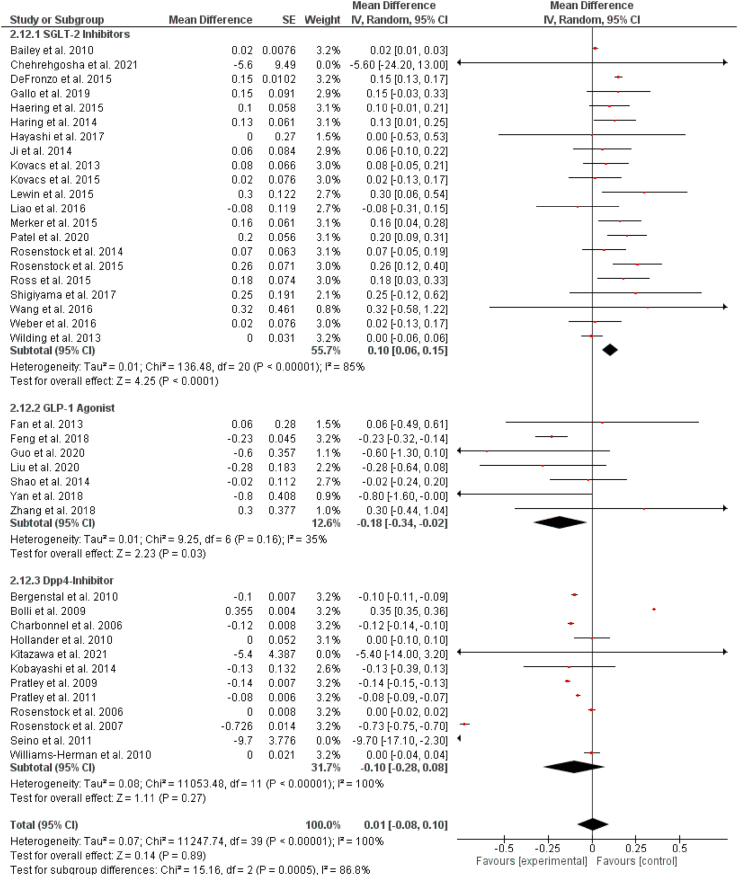
Pooled analysis of trials demonstrating effect of sodium glucose co-transport 2 inhibitors, glucagon-like peptide 1 agonist and dipeptidyl peptidase-4 inhibitors on total cholesterol.

Out of 57 selected studies, 21 reported the effect of SGLT-2 inhibitors on total cholesterol levels. Our pooled analysis demonstrates that SGLT-2 inhibitors significantly increase the levels of total cholesterol (WMD = 0.10 mg/dL, 95% CI: 0.06–0.15 mg/dL, P < 0.0001). Out of 57 selected studies, 7 reported the effect of GLP-1RAs on total cholesterol levels. Our results demonstrate that GLP-1RAs significantly reduce the levels of total cholesterol (WMD = −0.18 mg/dL, 95% CI: −0.34 to −0.02 mg/dL, P = 0.03). Out of 57 selected studies, 12 studies reported the effect of DPP-4 inhibitors on total cholesterol levels. We found that DPP-4 inhibitors had no significant effect on total cholesterol levels (WMD = −0.10 mg/dL, 95% CI: −0.28 to 0.08 mg/dL, P = 0.27). The results are summarized in Fig. 5.

### 3.6 Subgroup analysis for Type-2 diabetes mellitus

HDL: Out of 36 selected studies, 31 reported results on HDL for SGLT-2 inhibitors, 4 reported results for HDL on GLP-1RAs and only 1 study reported result for DPP4 inhibitors. Our pooled analysis demonstrated that SGLT-2 inhibitors had a significant impact in increasing HDL (WMD = 0.07 mg/dL, 95% CI: 0.06–0.08 mg/dL, P < 0.00001). However, GLP-1RAs had no significant effect on HDL levels (WMD = −0.02 mg/dL, 95% CI: −0.12 to 0.07 mg/dL, P = 0.64). In contrast, our analysis demonstrated that DPP4 inhibitors had a significant impact in increasing HDL (WMD = −6.00 mg/dL, 95% CI: −8.43 to −3.57 mg/dL, P < 0.00001). The results are summarized in Supplementary Fig. S1.

LDL: Out of 36 selected studies, 28 reported results on LDL for SGLT-2 inhibitors, 4 reported results for LDL on GLP-1RAs and only 1 study reported result for DPP4 inhibitors. Our pooled analysis demonstrated that SGLT-2 inhibitors had a significant role in increasing LDL (WMD = 0.12 mg/dL, 95% CI: 0.09–0.14 mg/dL, P < 0.00001). However, GLP-1RAs had no significant effect on LDL levels (WMD = 0.09 mg/dL, 95% CI: −0.08 to 0.26 mg/dL, P = 0.29). Similarly, DPP4 inhibitors had no significant effect on LDL levels (WMD = −2.00 mg/dL, 95% CI: −9.68 to 5.68 mg/dL, P = 0.61). The results are summarized in Supplementary Fig. S2.

Triglycerides: Out of 36 selected studies, 29 studies reported triglyceride results for SGLT-2 inhibitors, whereas only 1 study reported result for GLP-1RAs and 1 study reported results for DPP4 inhibitors. Our pooled analysis demonstrated that SGLT-2 inhibitors had a significant impact in decreasing triglyceride levels (WMD = −0.10 mg/dL, 95% CI: −0.13 to −0.06 mg/dL, P < 0.00001). In contrast, our analysis demonstrated that GLP1-RAs (WMD = −0.30 mg/dL, 95% CI: −0.80 to 0.20 mg/dL, P = 0.24) and DPP4 inhibitors (WMD = 9.80 mg/dL, 95% CI: −13.41 to 33.01 mg/dL, P = 0.41) did not show any significant changes in triglyceride levels. The results are summarized in Supplementary Fig. S3.

Total Cholesterol: Out of 36 selected studies, 19 studies reported total cholesterol results for SGLT-2 inhibitors, whereas only 1 study reported results for GLP-1RAs and 1 study reported results for DPP4 inhibitors. Our pooled analysis demonstrated that SGLT-2 inhibitors had a significant impact in increasing total cholesterol levels (WMD = 0.11 mg/dL, 95% CI: 0.06–0.16 mg/dL, P < 0.0001). In contrast, our analysis demonstrated that GLP1-RAs (WMD = 0.06 mg/dL, 95% CI: −0.49 to 0.61 mg/dL, P = 0.83) and DPP4 inhibitors (WMD = −5.40 mg/dL, 95% CI: −14.00 to 3.20 mg/dL, P = 0.22) did not show any significant changes in triglyceride levels. The results are summarized in Supplementary Fig. S4.

## 4 Discussion

We assess the effect of the three-novel glucose-lowering drug classes on hepatic fat parameters (HDL, LDL, TG, and total cholesterol) in this meta-analysis. Our combined findings demonstrate that SGLT-2 inhibitors were significantly associated with raising HDL, LDL, and cholesterol levels and reducing TG levels. The DPP-4 inhibitors class lower the level of triglycerides, and the GLP1-RA significantly reduces the levels of total cholesterol.

We discover that the three drug classes significantly reduce liver fat in NAFLD patients, which is similar to evidence from previous meta-analysis [11]. A comparative head-to-head meta-analysis was performed on 19 RCTs in only NAFLD patients to assess the effect of novel glucose-lowering treatments on liver parameters, including liver fat content which resulted in positive outcomes [11]. In a meta-analysis of seven RCTs, Mantovani et al. found that SGLT-2 inhibitors significantly improved liver fat content [28]. Another meta-analysis also assesses the effect of SGLT-2 inhibitors in NAFLD patients with T2DM, concluding that there is a significant decrease in liver fat content. However, the sample size of the studies was too small, there was a shorter follow-up time and this might have led to inhomogeneity of the results [29]. Another meta-analysis on incretin-based medicines (GLP1-RAs and DPP-4 inhibitors) indicates the efficacy of the agents in the treatment of NAFLD patients with concurrent T2DM. However, it failed to analyze hepatic fat endpoints or the SGLT-2 inhibitor medication class [3]. Thereby, no previous head-to-head study compares the potency of the three drug classes in NAFLD patients with T2DM and, more specifically, their effect on hepatic fat parameters (HDL, LDL, Triglycerides, and Cholesterol). All the previously conducted meta-analyses had certain limitations regarding included studies, follow-up time, prognostic indicators, and study population (only NAFLD patients, regional/ethnic bias, and the presence of specific co-morbidities like hypertension), which may mask the actual outcome. Moreover, the studies either have only NAFLD patients as the target population or only evaluate the effect of SGLT-2 inhibitors, GLP-1RAs agonists and DPP-4 inhibitors on liver enzymes. Hence, this updated meta-analysis fills this knowledge gap and comprehensively evaluates the impact of the three-novel glucose-lowering drug classes on hepatic fat parameters in T2DM patients with or without NAFLD.

Our subgroup analysis on type 2 DM patients demonstrated that SGLT-2 inhibitors had a significant role in increasing HDL and LDL from baseline. SGLT-2 inhibitors significantly reduced triglyceride levels, however, they also increased total cholesterol. On the other hand, GLP-1RAs had no influence on the lipid profiles. A previous meta-analysis on GLP-1RAs has demonstrated similar results on HDL, where overall GLP-1RAs demonstrated beneficial effects on HDL, however, our analysis demonstrates opposite results [30]. A difference in our subgroup analysis may be due to inclusion of trials only with patients with T2DM who did not have any other comorbidities, but the previous meta-analysis included participants with other comorbidities as well.

SGLT-2 inhibitors are a unique class of diabetes medications authorized to treat T2DM. These medications work by impairing the kidney's capability of absorbing filtered glucose. SGLT-2 inhibitors boost renal glycosuria and osmotic diuresis, improving glucose management, increase weight loss, and lead to blood pressure reduction. Because SGLT-2 inhibitors enhance glucose management, body weight, and blood pressure, they may improve the prognosis of individuals with T2DM who have concurrent NAFLD [28].

Glucoregulatory action of GLP-1 incretin peptide is a distinct category of treatments for T2DM therapy. GLP-1 is released in the distal ileum and proximal colon by L-cells. It binds to pancreatic, renal, pulmonary, gastrointestinal, and peripheral nervous system receptors. GLP-1 stimulates insulin production, inhibits glucagon release, delays stomach emptying, inhibits glucagon release, and produces glucose. NAFLD patients may benefit from using GLP-1RAs since they help create early satiety, decrease hunger, and delay stomach emptying, thereby contributing to weight loss [3]. Aside from its weight-loss benefits, GLP-1RAs also substantially influence hepatic lipid content via increasing insulin signaling pathways and fatty acid utilization (3). An enzyme called DPP-4 rapidly degrades GLP-1, which DPP-4 inhibitors target. This process serves to maintain the incretin peptide GLP functioning for a longer time. This process would undoubtedly improve the prognosis of NAFLD patients [3].

Few studies looked at how DPP-4 inhibitors affect LDL levels, therefore making it vital that future trials are designed to elucidate the full influence of DPP-4 inhibitors on LDL levels. In our study, only three trials assessed the impact of DPP-4 inhibitors on LDL levels in NAFLD patients. Specifically, the effect of GLP-1RA's on TG needs further investigation. Only four trials in our study evaluated the impact of GLP-1RA's on TG, making more studies on this topic necessary. GLP-1RAs and DPP-4 inhibitors were assessed in only 1 study for subgroup analysis on patients with T2DM, therefore more trials including patients with only T2DM without any comorbidities should be conducted to further improve the analysis and compare the effect of three-novel glucose agents for patients with T2DM better. Few studies assessing the effects of these drugs on lipid parameters becomes an obstacle for obtaining valid and rigorous results that can improve the guidelines for the patients.

The main strengths of our study include the inclusion of a large number of studies given the novelty of this topic and hence, a sizeable sample of the patient population, thereby, increasing the statistical power of our analysis, systematic literature search with well-defined inclusion criteria and lastly, detailed data extraction. More importantly, our study is the first with a head-to-head analysis assessing the effects of the 3-novel glucose lowering drugs on hepatic fat parameters in NAFLD patients with and without T2DM, hence, making a significant contribution in the literature and opening new doors for research in this context. Lastly, we even conducted a sub-group analysis incorporating patients with only T2DM and so evaluating the effectiveness of drugs in only diabetic patients, not conducted previously either.

Our study does have limitations. We identified heterogeneity among the included trials; hence, the findings should be interpreted cautiously. In the future, more head-to-head studies are required with newer trials and a sensitivity analysis, assessing the change in heterogeneity by removing the trials with high risk of bias, or performing a leave-one out sensitivity analysis. Our research opens up paths to more rigorous analysis to compare the best drugs in the three-novel glucose lowering drug classes and then comparing them in head-to-head study design. The heterogeneity in the trials exists because they vary in their primary endpoint, treatment duration, comparator, concomitant medication, and inclusion criteria; these factors and may lead to bias. Secondly, some of the patients had only NAFLD without T2DM, which could also create a discrepancy in the results. To address methodological heterogeneity, we performed our study using a random-effects model. Thus, estimates derived from our analysis should not be considered definitive, however, they should be taken as a guide to what benefits could be expected in treatment with the three-novel glucose lowering agents and hence, a subgroup analysis was not performed on the follow-up duration of the studies. The follow-up time in some studies was also too short. Future studies would need longer follow up times to better gauge the effects of the glucose lowering agents on hepatic fat content.

In conclusion, our meta-analysis is the most comprehensive and updated assessment of a placebo-controlled, or head-to-head RCTs of individuals with T2DM with or without NAFLD using the 3-novel glucose-lowering drug classes. Our study demonstrates a significant rise in HDL, LDL, and cholesterol by SGLT-2 inhibitors. We identify a decrease in TG content by DPP-4 inhibitors, and a significant reduction in cholesterol by GLP-1RA's. From these conclusions, this paper adds to T2DM literature and provides indispensable insights into a new chapter of potential pharmacotherapies for its treatment. Since there was a paucity in data evaluating the DPP-4 inhibitor drug class, from here on, more research is requisite for the DPP-4 inhibitor class in patients with T2DM without any comorbidities. This finding is also essential for physicians as our study demonstrated SGLT2 inhibitors having significant impact in diabetic patients, however, in future the clinicians and patients would benefit much more if a rigorous analysis for other 2 drug classes have also been performed. Lastly, more extensive studies and randomized placebo-controlled trials with a longer duration of follow-up are necessary to build a robust collection of evidence about the effects of the three-novel glucose-lowering drug classes on hepatic fat parameters in T2DM patients with or without NAFLD.

## Ethical approval

None.

## Sources of funding for your research

None.

## Author contributions

Sophia Dar (study conception and design; writing the paper).

Ahmed Kamal Siddiqi (writing the paper; data collection).

Tamim Omar Alabduladhem (writing the paper).

Ahmed Mustafa Rashid (writing the paper).

Saba Sarfraz (data collection; data analysis).

Talha Maniya (data analysis).

Ritesh G. Menezes (data interpretation).

Talal Almas (data interpretation).

## Registration of research studies

Name of the registry: None
Unique Identifying number or registration ID: None
Hyperlink to your specific registration (must be publicly accessible and will be checked): None

## Consent

None.

## Guarantor

Talal Almas.

## Provenance and peer review

Not commissioned, externally peer-reviewed.

## Declaration of competing interest

None.
