# Supplementary material for: Effects of novel glucose-lowering drugs on the lipid parameters: A systematic review and meta-analysis
Source: Ann Med Surg (Lond). 2022 Apr 16;77:103633. doi: 10.1016/j.amsu.2022.103633 (PMC9142616; doi:10.1016/j.amsu.2022.103633)
Supplement: Multimedia component 1 [file mmc1.docx]

**Supplementary Table 1**

| ("SGLT-2"[All Fields] OR "sglt2"[All Fields] OR ("canagliflozin"[MeSH Terms] OR "canagliflozin"[All Fields]) OR ("canagliflozin"[MeSH Terms] OR "canagliflozin"[All Fields] OR "invokana"[All Fields]) OR ("dapagliflozin"[Supplementary Concept] OR "dapagliflozin"[All Fields] OR "dapagliflozin s"[All Fields]) OR ("dapagliflozin propanediol"[All Fields] OR "dapagliflozin"[Supplementary Concept] OR "dapagliflozin"[All Fields] OR "farxiga"[All Fields] OR "dapagliflozin s"[All Fields]) OR ("empagliflozin"[Supplementary Concept] OR "empagliflozin"[All Fields]) OR ("empagliflozin"[Supplementary Concept] OR "empagliflozin"[All Fields] OR "jardiance"[All Fields])) AND ((("lipid s"[All Fields] OR "lipidate"[All Fields] OR "lipidated"[All Fields] OR "lipidates"[All Fields] OR "lipidation"[All Fields] OR "lipidations"[All Fields] OR "lipide"[All Fields] OR "lipides"[All Fields] OR "lipidic"[All Fields] OR "lipids"[MeSH Terms] OR "lipids"[All Fields] OR "lipid"[All Fields]) AND ("parameter"[All Fields] OR "parameter s"[All Fields] OR "parameters"[All Fields])) OR ("oxidized low density lipoprotein"[Supplementary Concept] OR "oxidized low density lipoprotein"[All Fields] OR "ldl"[All Fields]) OR "HDL"[All Fields] OR ("triglycerid"[All Fields] OR "triglycerides"[MeSH Terms] OR "triglycerides"[All Fields] OR "triglyceride"[All Fields] OR "triglycerids"[All Fields]) OR (("total"[All Fields] OR "totaled"[All Fields] OR "totaling"[All Fields] OR "totalled"[All Fields] OR "totalling"[All Fields] OR "totals"[All Fields]) AND ("cholesterol"[MeSH Terms] OR "cholesterol"[All Fields] OR "cholesterol s"[All Fields] OR "cholesterole"[All Fields] OR "cholesterols"[All Fields]))) |
| --- |

| **study ID** | **Study Design** | **Target Population** | **sample size** | **intervention** | **comparator** | **follow-up** |
| --- | --- | --- | --- | --- | --- | --- |
| Aronson et al. 2018 (1) | Randomized double-blind controlled trial | Type 2 diabetes | 305 | Ertugliflozin 15mg | placebo | 52 weeks |
| Bailey et al. 2010 | Randomized, Double-Blind, Placebo-Controlled Clinical Trial | Type 2 diabetes | 266 | Dapagliflozin 10 mg | placebo | 24 weeks |
| Bergenstal et al. 2010 | Randomized controlled trial | DM | 331 | Sitagliptin | Pioglitazone | 260 weeks |
| Bode B. et al. 2013 | Randomized double-blind controlled trial | Type 2 diabetes | 714 | Canagliflozin 300mg | placebo | 26 weeks |
| Bolli et al. 2009 | Randomized, active controlled trial | DM | 576 | Vildagliptin | Piogitazone | 312 weeks |
| Bouchi et al. 2016 | Randomized open‐label controlled trial | Type 2 diabetes | 17 | liraglutide + insulin | insulin | 12 weeks |
| Cefalu et al. 2013 | Randomized,  double-blind, active-  controlled trial | Type 2 diabetes | 967 | Canagliflozin  300 mg | Glimepiride 1.8mg | 52 weeks |
| Charbonnel et al. 2006 | randomized, parallel-group study with a placebo controlled, double-blind | DM | 701 | Sitagliptin | placebo | 312 weeks |
| Chehrehgosha et al. 2021 | Randomized, Double-Blind, Placebo-Controlled Clinical Trial | Type 2 diabetes | 72 | empagliflozin | placebo | 24 weeks |
| DeFronzo et al. 2015 | Randomized, double-blind, active-controlled | Type 2 diabetes | 268 | empagliflozin | linagliptin | 52 weeks |
| Fan et al. 2013 | Randomized controlled trial | Type 2 diabetes | 117 | Exenatide | Metformin | 12 weeks |
| Feng et al. 2018 | Randomized,  double-blind,  placebo-controlled trial | Type 2 diabetes with MAFLD | 58 | Liraglutide | Metformin | 24 weeks |
| Forst et  al. 2014 | Randomized, double-blind, placebo-controlled | Type 2 diabetes | 229 | Canagliflozin | placebo | 26 weeks |
| Gallo et al. 2019 | Randomized, double-blind, placebo-controlled | Type 2 diabetes | 414 | Ertugliflozin 15mg | placebo | 104 weeks |
| Guo et al. 2020 | Randomized placebo-controlled trial | Type 2 diabetes and obesity | 61 | Liraglutide | Placebo | 26 weeks |
| Haering et al. 2015 | Randomized, double-blind, placebo-controlled | Type 2 diabetes | 441 | Empagliflozin 25 mg | placebo | 76 weeks |
| Häring et al. 2014 | Randomized, double-blind, placebo-controlled | Type 2 diabetes | 441 | Empagliflozin 25 mg | placebo | 24 weeks |
| Hayashi et al. 2017 | Randomized open‐label controlled trial | Type 2 diabetes | 80 | Dapagliflozin 5mg | Sitagliptin 50 mg | 12 weeks |
| Hollander et al. 2010 | Randomized, three-arm,double-blind, placebo-controlled trial | DM | 565 | Sitagliptin | placebo | 260 weeks |
| Inagaki et al. 2014 | Randomized, double-blind, placebo-controlled | Type 2 diabetes | 181 | canagliflozin | placebo | 24 weeks |
| Inagaki et al. 2016 | Randomized, double-blind, placebo-controlled | Type 2 diabetes | 146 | canagliflozin | placebo | 16 weeks |
| Ji et al. 2014 | Randomized, double-blind, placebo-controlled | Type 2 diabetes | 265 | Dapagliflozin 10 mg | placebo | 24 weeks |
| Kashiwagi et al. 2018 | Randomized,  double-blind,  placebo-controlled trial | Type 2 diabetes | 996 | Ipragliflozin 50mg | placebo | 12-24 weeks |
| Kitazawa et al. 2020 | Randomized, active-  controlled, open-  label trial | Type 2 diabetes | 64 | Tofogliflozin 20mg | Glimepiride 0.5mg | 24 weeks |
| Kobayashi et al. 2014 | open-label, double blind randomized controlled | DM | 114 | sitagliptin | gluscosidase inhibitor | 24 weeks |
| Kovacs et al. 2013 | Randomized, double-blind, placebo-controlled | Type 2 diabetes | 333 | Empagliflozin 25 mg | placebo | 24 weeks |
| Kovacs et al. 2015 | Randomized, double-blind, placebo-controlled | Type 2 diabetes | 333 | Empagliflozin 25 mg | placebo | 76 weeks |
| Kuchay et al. 2020 | Randomized,  double-blind,  placebo-controlled trial | Type 2 diabetes | 64 | Dulaglutide and standard treatment for type 2 diabetes | standard treatment for type 2 diabetes | 24 weeks |
| Lavelle- Gonzalez et al. 2013 | Randomized double-blind controlled trial | Type 2 diabetes | 733 | Canagliflozin 300mg | Sitagliptin 50 mg | 52 weeks |
| Leiter et al. 2015 | Randomized, double-blind, active-controlled | Type 2 diabetes | 967 | Canagliflozin | Glimepiride | 104 weeks |
| Lewin et al. 2015 | Randomized, double-blind, active-controlled | Type 2 diabetes | 266 | empagliflozin | Linagliptin | 52 weeks |
| Liao et al. 2016 | Randomized,  double-blind,  placebo-controlled trial | Type 2 diabetes | 162 | Dapagliflozin 10mg | placebo | 12 weeks |
| Liu et al. 2020 | Randomized controlled trial | Type 2 diabetes with MAFLD | 74 | Exenatide | Insulin glargine | 24 weeks |
| Matikainen et al. 2018 | single‐centre randomized controlled | Type 2 diabetes | 22 | liraglutide | placebo | 16 weeks |
| Merker et al. 2015 | Randomized, double-blind, placebo-controlled | Type 2 diabetes | 420 | Empagliflozin | placebo | 24 weeks |
| Patel et al. 2020 | Randomized, double-blind, placebo-controlled | Type 2 diabetes | 1029 | Empagliflozin | placebo | 26 weeks |
| Pratley et al. 2009 | double-blind, randomized, placebo-con- trolled | DM | 312 | Alogliptin | placebo | 312 weeks |
| Pratley et al. 2011 | randomised, parallel-group, open-label trial | DM | 665 | Sitagliptin | liraglutide | 312 weeks |
| Rosenstock et al. 2006 | randomized,  double-blind, placebo-controlled, parallel-group | DM | 353 | Sitagliptin | placebo | 312 weeks |
| Rosenstock et al. 2007 | double-blind, randomized, active- controlled, parallel-group | DM | 453 | vildagliptin | placebo | 104 weeks |
| Rosenstock et al. 2014 | Randomized, double-blind, placebo-controlled | Type 2 diabetes | 377 | Empagliflozin | placebo | 52 weeks |
| Rosenstock et al. 2015 | Randomized, double-blind, placebo-controlled | Type 2 diabetes | 325 | Empagliflozin | placebo | 78 weeks |
| Ross et al. 2015 | Randomized, placebo-controlled | Type 2 diabetes | 325 | Empagliflozin | placebo | 16 weeks |
| Schernthaner G et al. 2013 | Randomized double-blind controlled trial | Type 2 diabetes | 755 | Canagliflozin 300mg | Sitagliptin 50 mg | 52 weeks |
| Seino et al. 2011 | randomized, double-blind, parallel group | DM | 162 | Alogliptin | voglibose | 12 weeks |
| Shao et al. 2014 | Randomized controlled trial | Type 2 diabetes with MAFLD | 60 | Exenatide | Insulin | 12 weeks |
| Shigiyama et al. 2017 | Randomized, open-label, blinded-endpoint | Type 2 diabetes | 80 | Dapagliflozin | Metformin | 16 weeks |
| Shimizu et al. 2019 | Randomized double-blind controlled trial | Type 2 diabetes | 63 | Dapagliflozin 5mg | standard therapy without SGLT2 inhibitors | 24 weeks |
| Stenlof et al. 2013 | Randomized, double-blind, placebo-controlled | Type 2 diabetes | 387 | Canagliflozin | placebo | 26 weeks |
| Tang et al. 2015 | Randomized controlled trial | Type 2 diabetes with MAFLD | 35 | Liraglutide | Insulin | 12 weeks |
| Wang et al. 2016 | Randomized clinical trial | Type 2 diabetes and hypertension | 28 | Dapagliflozin | placebo | 24 weeks |
| Weber et al. 2016 | Randomized, double-blind, placebo-controlled | Type 2 diabetes and hypertension | 641 | Canagliflozin | placebo | 12 weeks |
| Wilding et al. 2013 | Randomized, double-blind, placebo-controlled | Type 2 diabetes | 312 | Dapagliflozin | placebo | 52 weeks |
| Williams-Herman et al. 2010 | Randomized, double blind, controlled trial | DM | 330 | Sitagliptin | placebo | 208 weeks |
| Yale et al. 2013 | Randomized, double-blind, placebo-controlled | Type 2 diabetes and chronic kidney disease | 179 | Canagliflozin | placebo | 52 weeks |
| Yan et al. 2018 | Randomized controlled trial | Type 2 diabetes with MAFLD | 48 | Liraglutide | Insulin | 26 weeks |
| Zhang et al. 2018 | Randomized controlled trial | Type 2 diabetes with MAFLD | 60 | Liraglutide | Pioglitazone | 24 weeks |

**Supplementary table 2**

|  |  |  |  |  |  |  |  |
| --- | --- | --- | --- | --- | --- | --- | --- |
| **Authors** | **Random sequence  generation  (selection bias)** | **Allocation  concealment  (selection bias)** | **Blinding of  participants and  personnel  (performance  bias)** | **Blinding of  outcome  assessment  (detection  bias)** | **Incomplete  outcome  data  (attrition  bias)** | **Selective  reporting  (reporting  bias)** | **Other  Bias *** |
| Aronson et al. 2018 | L | L | L | L | L | L | L |
| Bailey et al. 2010 | L | L | L | L | L | L | L |
| Bergenstal et al. 2010 | L | L | L | L | L | L | L |
| Bode B. et al. 2013 | L | L | L | L | L | L | L |
| Bolli et al. 2009 | L | L | L | L | L | L | L |
| Bouchi et al. 2016 | L | U | U | U | U | U | U |
| Cefalu et al. 2013 | L | L | L | L | L | L | L |
| Charbonnel et al. 2006 | H | U | U | U | L | L | U |
| Chehrehgosha et al. 2021 | L | L | L | L | L | L | L |
| DeFronzo et al. 2015 | L | L | U | U | L | L | L |
| Fan et al. 2013 | U | U | H | L | L | U | L |
| Feng et al. 2018 | L | L | L | L | L | L | L |
| Forst et al. 2014 | L | L | L | L | L | L | L |
| Gallo et al. 2019 | U | U | U | U | L | L | U |
| Guo et al. 2020 | L | U | H | H | U | U | U |
| Haering et al. 2015 | L | L | U | U | L | L | U |
| Häring et al. 2014 | L | L | U | U | L | L | U |
| Hayashi et al. 2017 | U | U | H | H | L | L | U |
| Hollander et al. 2010 | L | U | U | U | L | L | U |
| Inagaki et al. 2014 | L | L | U | U | L | L | U |
| Inagaki et al. 2016 | L | U | U | U | L | L | U |
| Ji et al. 2014 | L | L | L | L | L | L | L |
| Kashiwagi et al. 2018 | U | U | U | U | L | L | U |
| Kitazawa et al. 2020 | U | U | H | H | L | L | U |
| Kobayashi et al. 2014 | U | U | U | U | U | U | U |
| Kovacs et al. 2013 | L | L | U | U | L | L | L |
| Kovacs et al. 2015 | L | L | U | U | L | L | L |
| Kuchay et al. 2020 | L | L | L | H | L | L | U |
| Lavelle- Gonzalez et al. 2013 | L | U | L | L | L | L | L |
| Leiter et al. 2015 | L | U | L | L | L | L | L |
| Lewin et al. 2015 | L | U | L | L | U | L | L |
| Liao et al. 2016 | L | L | L | L | L | L | L |
| Liu et al. 2020 | L | U | H | U | U | L | L |
| Matikainen et al. 2018 | L | U | U | U | U | U | U |
| Merker et al. 2015 | L | L | L | L | L | L | U |
| Patel et al. 2020 | U | U | L | L | L | L | U |
| Pratley et al. 2009 | U | U | U | U | U | U | U |
| Pratley et al. 2011 | U | U | U | U | U | U | U |
| Rosenstock et al. 2006 | H | U | U | U | L | L | U |
| Rosenstock et al. 2007 | U | U | U | U | U | U | U |
| Rosenstock et al. 2014 | L | U | L | L | L | L | L |
| Rosenstock et al. 2015 | L | U | L | L | L | L | L |
| Ross et al. 2015 | L | U | U | U | L | L | L |
| Schernthaner G et al. 2013 | L | L | L | L | L | L | L |
| Seino et al. 2011 | L | L | L | L | L | L | L |
| Shao et al. 2014 | L | U | U | L | L | U | L |
| Shigiyama et al. 2017 | L | U | H | H | L | L | L |
| Shimizu et al. 2019 | L | U | H | H | L | L | U |
| Stenlof et al. 2013 | L | U | L | L | L | L | L |
| Tang et al. 2015 | L | U | L | L | L | L | L |
| Wang et al. 2016 | L | U | U | U | L | L | U |
| Weber et al. 2016 | L | U | L | L | L | L | L |
| Wilding et al. 2013 | L | U | L | L | L | L | L |
| Williams-Herman et al. 2010 | L | U | U | U | L | L | U |
| Yale et al. 2013 | L | U | L | L | L | L | L |
| Yan et al. 2018 | L | U | L | U | L | L | L |
| Zhang et al. 2018 | L | U | H | U | L | U | L |

**Supplementary table 3**

**Figure legends:**

**Supplementary Figure S1:** Subgroup analysis of trials demonstrating effect of sodium glucose co-transport 2 inhibitors, glucagon-like peptide 1 agonist and dipeptidyl peptidase-4 inhibitors on HDL in patients with Type 2 diabetes mellitus

**Supplementary Figure S2:** Subgroup analysis of trials demonstrating effect of sodium glucose co-transport 2 inhibitors, glucagon-like peptide 1 agonist and dipeptidyl peptidase-4 inhibitors on LDL in patients with Type 2 diabetes mellitus

**Supplementary Figure S3:** Subgroup analysis of trials demonstrating effect of sodium glucose co-transport 2 inhibitors, glucagon-like peptide 1 agonist and dipeptidyl peptidase-4 inhibitors on triglyceride levels in patients with Type 2 diabetes mellitus

**Supplementary Figure S1:** Subgroup analysis of trials demonstrating effect of sodium glucose co-transport 2 inhibitors, glucagon-like peptide 1 agonist and dipeptidyl peptidase-4 inhibitors on total cholesterol levels in patients with Type 2 diabetes mellitus

**Supplementary Figure S1**

**
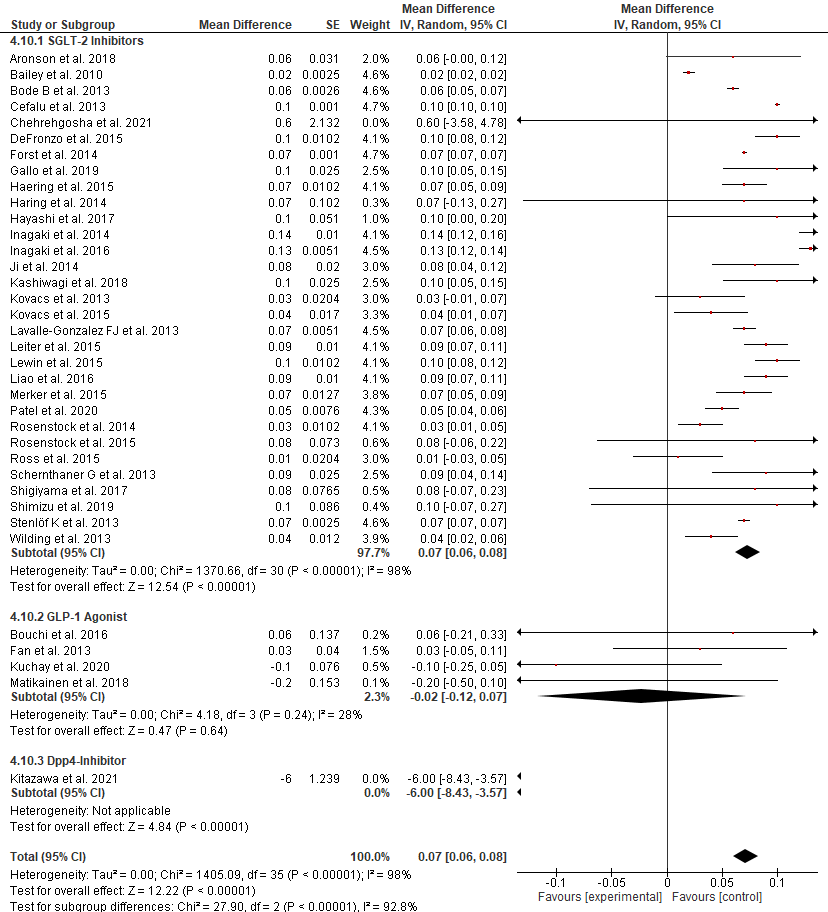
**

**Supplementary Figure S2**

**
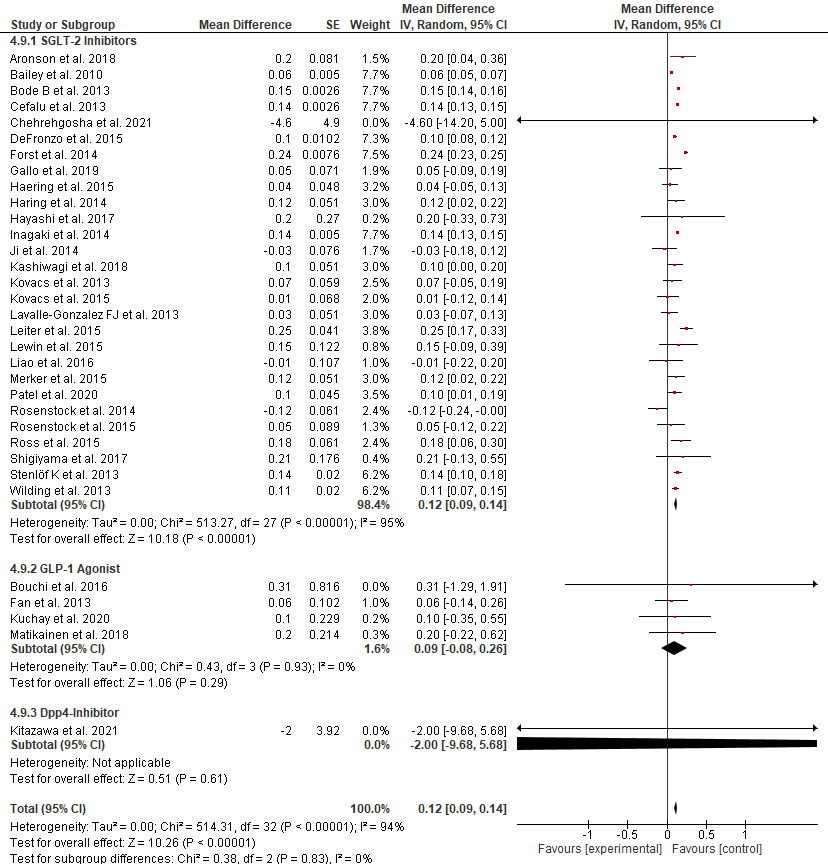
**

**Supplementary Figure S3**

**
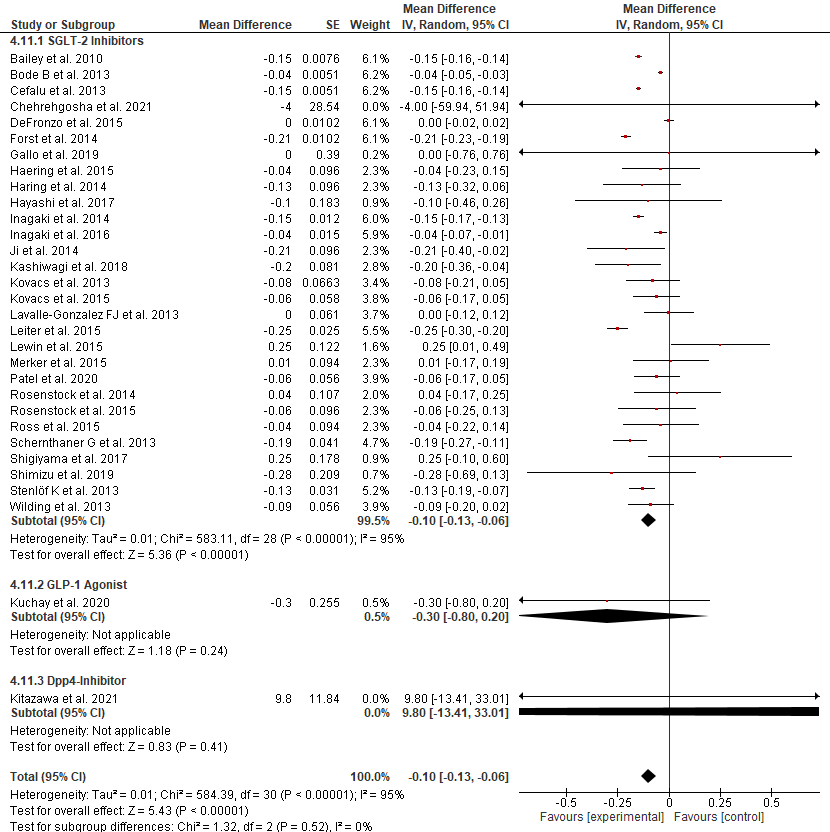
**

**Supplementary Figure S4**

**
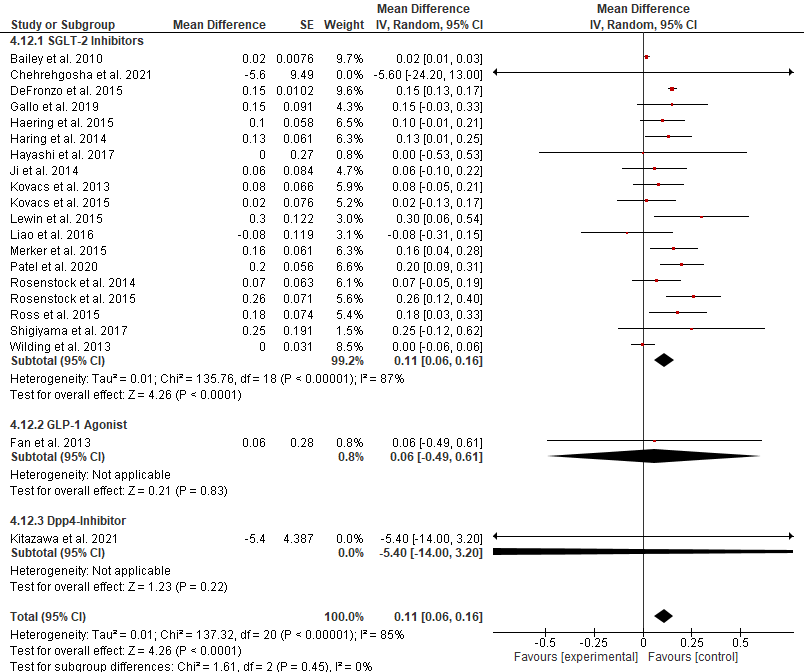
**

**References:**

1. Aronson R, Frias J, Goldman A, Darekar A, Lauring B, Terra SG. Long‐term efficacy and safety of ertugliflozin monotherapy in patients with inadequately controlled T2DM despite diet and exercise: VERTIS MONO extension study. Diabetes, Obesity and Metabolism. 2018 Jun;20(6):1453-60.
2. Bailey CJ, Gross JL, Pieters A, Bastien A, List JF. Effect of dapagliflozin in patients with type 2 diabetes who have inadequate glycaemic control with metformin: a randomised, double-blind, placebo-controlled trial. The Lancet. 2010 Jun 26;375(9733):2223-33.
3. Bergenstal RM, Wysham C, MacConell L, Malloy J, Walsh B, Yan P, Wilhelm K, Malone J, Porter LE, DURATION-2 Study Group. Efficacy and safety of exenatide once weekly versus sitagliptin or pioglitazone as an adjunct to metformin for treatment of type 2 diabetes (DURATION-2): a randomised trial. The Lancet. 2010 Aug 7;376(9739):431-9.
4. Bode B, Stenlöf K, Sullivan D, Fung A, Usiskin K. Efficacy and safety of canagliflozin treatment in older subjects with type 2 diabetes mellitus: a randomized trial. Hospital practice. 2013 Apr 1;41(2):72-84.
5. Bolli G, Dotta F, Colin L, Minic B, Goodman M. Comparison of vildagliptin and pioglitazone in patients with type 2 diabetes inadequately controlled with metformin. Diabetes, Obesity and Metabolism. 2009 Jun;11(6):589-95.
6. Bouchi R, Nakano Y, Fukuda T, Takeuchi T, Murakami M, Minami I, Izumiyama H, Hashimoto K, Yoshimoto T, Ogawa Y. Reduction of visceral fat by liraglutide is associated with ameliorations of hepatic steatosis, albuminuria, and micro-inflammation in type 2 diabetic patients with insulin treatment: a randomized control trial. Endocrine journal. 2016:EJ16-0449.
7. Cefalu WT, Leiter LA, Yoon KH, Arias P, Niskanen L, Xie J, Balis DA, Canovatchel W, Meininger G. Efficacy and safety of canagliflozin versus glimepiride in patients with type 2 diabetes inadequately controlled with metformin (CANTATA-SU): 52 week results from a randomised, double-blind, phase 3 non-inferiority trial. Lancet. 2013;382(9896):941-50. doi: 10.1016/S0140-6736(13)60683-2.
8. Charbonnel B, Karasik A, Liu J, Wu M, Meininger G. Efficacy and safety of the dipeptidyl peptidase-4 inhibitor sitagliptin added to ongoing metformin therapy in patients with type 2 diabetes inadequately controlled with metformin alone. Diabetes care. 2006 Dec 1;29(12):2638-43.
9. Chehrehgosha H, Sohrabi MR, Ismail-Beigi F, et al. Empagliflozin Improves Liver Steatosis and Fibrosis in Patients with Non-Alcoholic Fatty Liver Disease and Type 2 Diabetes: A Randomized, Double-Blind, Placebo-Controlled Clinical Trial. Diabetes Ther. 2021;12(3):843-861. doi:10.1007/s13300-021-01011-3
10. DeFronzo RA, Lewin A, Patel S, Liu D, Kaste R, Woerle HJ, Broedl UC. Combination of empagliflozin and linagliptin as second-line therapy in subjects with type 2 diabetes inadequately controlled on metformin. Diabetes care. 2015 Mar 1;38(3):384-93.
11. Fan H, Pan Q, Xu Y, Yang X. Exenatide improves type 2 diabetes concomitant with nonalcoholic fatty liver disease. Arq Bras Endocrinol Metabol. 2013 Dec;57(9):702-8. doi: 10.1590/s0004-27302013000900005. PMID: 24402015.
12. Feng WH, Bi Y, Li P, Yin TT, Gao CX, Shen SM, Gao LJ, Yang DH, Zhu DL. Effects of liraglutide, metformin and gliclazide on body composition in patients with both type 2 diabetes and non-alcoholic fatty liver disease: A randomized trial. J Diabetes Investig. 2019 Mar;10(2):399-407. doi: 10.1111/jdi.12888. Epub 2018 Aug 16. PMID: 29957886; PMCID: PMC6400178.
13. Forst T, Guthrie R, Goldenberg R, Yee J, Vijapurkar U, Meininger G, Stein P. Efficacy and safety of canagliflozin over 52 weeks in patients with type 2 diabetes on background metformin and pioglitazone. Diabetes, Obesity and Metabolism. 2014 May;16(5):467-77.
14. Gallo S, Charbonnel B, Goldman A, Shi H, Huyck S, Darekar A, Lauring B, Terra SG. Long‐term efficacy and safety of ertugliflozin in patients with type 2 diabetes mellitus inadequately controlled with metformin monotherapy: 104‐week VERTIS MET trial. Diabetes, Obesity and Metabolism. 2019 Apr;21(4):1027-36.
15. Guo W, Tian W, Lin L, Xu X. Liraglutide or insulin glargine treatments improves hepatic fat in obese patients with type 2 diabetes and nonalcoholic fatty liver disease in twentysix weeks: A randomized placebo-controlled trial. Diabetes Res Clin Pract. 2020 Dec;170:108487. doi: 10.1016/j.diabres.2020.108487. Epub 2020 Oct 6. PMID: 33035599.
16. Häring HU, Merker L, Seewaldt-Becker E, Weimer M, Meinicke T, Woerle HJ, Broedl UC, EMPA-REG METSU Trial Investigators. Empagliflozin as add-on to metformin plus sulfonylurea in patients with type 2 diabetes: a 24-week, randomized, double-blind, placebo-controlled trial. Diabetes care. 2013 Nov 1;36(11):3396-404.
17. Häring HU, Merker L, Seewaldt-Becker E, Weimer M, Meinicke T, Woerle HJ, Broedl UC, EMPA-REG METSU Trial Investigators. Empagliflozin as add-on to metformin plus sulfonylurea in patients with type 2 diabetes: a 24-week, randomized, double-blind, placebo-controlled trial. Diabetes care. 2013 Nov 1;36(11):3396-404.
18. Hayashi T, Fukui T, Nakanishi N, Yamamoto S, Tomoyasu M, Osamura A, Ohara M, Yamamoto T, Ito Y, Hirano T. Dapagliflozin decreases small dense low-density lipoprotein- cholesterol and increases high-density lipoprotein 2-cholesterol in patients with type 2 diabetes: comparison with sitagliptin. Cardiovasc Diabetol. 2017;16(1):8. doi: 10.1186/s12933-016-0491-5
19. Hollander P, Li J, Allen E, Chen R, CV181-013 Investigators. Saxagliptin added to a thiazolidinedione improves glycemic control in patients with type 2 diabetes and inadequate control on thiazolidinedione alone. The Journal of Clinical Endocrinology & Metabolism. 2009 Dec 1;94(12):4810-9.
20. Inagaki N, Kondo K, Yoshinari T, Takahashi N, Susuta Y, Kuki H. Efficacy and safety of canagliflozin monotherapy in Japanese patients with type 2 diabetes inadequately controlled with diet and exercise: a 24-week, randomized, double-blind, placebo-controlled, Phase III study. Expert opinion on pharmacotherapy. 2014 Aug 1;15(11):1501-15.
21. Inagaki N, Harashima SI, Maruyama N, Kawaguchi Y, Goda M, Iijima H. Efficacy and safety of canagliflozin in combination with insulin: a double-blind, randomized, placebo-controlled study in Japanese patients with type 2 diabetes mellitus. Cardiovascular diabetology. 2016 Dec;15(1):1-2.
22. Ji L, Ma J, Li H, Mansfield TA, T’joen CL, Iqbal N, Ptaszynska A, List JF. Dapagliflozin as monotherapy in drug-naive Asian patients with type 2 diabetes mellitus: a randomized, blinded, prospective phase III study. Clinical therapeutics. 2014 Jan 1;36(1):84-100.
23. Kashiwagi A, Sakatani T, Nakamura I, Akiyama N, Kazuta K, Ueyama E, Takahashi H, Kosakai Y. Improved cardiometabolic risk factors in Japanese patients with type 2 diabetes treated with ipragliflozin: a pooled analysis of six randomized, placebocontrolled trials. Endocr J. 2018;65(7):693-705. doi: 10.1507/endocrj.EJ17-0491.
24. Kitazawa T, Seino H, Ohashi H, et al. Comparison of tofogliflozin versus glimepiride as the third oral agent added to metformin plus a dipeptidyl peptidase-4 inhibitor in Japanese patients with type 2 diabetes: A randomized, 24-week, open-label, controlled trial (STOP- OB). Diabetes Obes Metab. 2020;10.1111/dom.14059. doi:10.1111/dom.14059.
25. Kobayashi K, Yokoh H, Sato Y, Takemoto M, Uchida D, Kanatsuka A, Kuribayashi N, Terano T, Hashimoto N, Sakurai K, Hanaoka H. Efficacy and safety of the dipeptidyl peptidase‐4 inhibitor sitagliptin compared with α‐glucosidase inhibitor in Japanese patients with type 2 diabetes inadequately controlled on sulfonylurea alone (SUCCESS‐2): a multicenter, randomized, open‐label, non‐inferiority trial. Diabetes, Obesity and Metabolism. 2014 Aug;16(8):761-5.
26. Kovacs CS, Seshiah V, Swallow R, Jones R, Rattunde H, Woerle HJ, Broedl UC, EMPA‐REG PIO™ trial investigators. Empagliflozin improves glycaemic and weight control as add‐on therapy to pioglitazone or pioglitazone plus metformin in patients with type 2 diabetes: a 24‐week, randomized, placebo‐controlled trial. Diabetes, Obesity and Metabolism. 2014 Feb;16(2):147-58.
27. Kovacs CS, Seshiah V, Merker L, Christiansen AV, Roux F, Salsali A, Kim G, Stella P, Woerle HJ, Broedl UC. Empagliflozin as add-on therapy to pioglitazone with or without metformin in patients with type 2 diabetes mellitus. Clinical therapeutics. 2015 Aug 1;37(8):1773-88.
28. Kuchay MS, Krishan S, Mishra SK, Choudhary NS, Singh MK, Wasir JS, Kaur P, Gill HK, Bano T, Farooqui KJ, Mithal A. Effect of dulaglutide on liver fat in patients with type 2 diabetes and NAFLD: randomised controlled trial (D-LIFT trial). Diabetologia. 2020 Nov;63(11):2434-2445. doi: 10.1007/s00125-020-05265-7. Epub 2020 Aug 31. PMID: 32865597.
29. Lavalle-González FJ, Januszewicz A, Davidson J, Tong C, Qiu R, Canovatchel W, Meininger G. Efficacy and safety of canagliflozin compared with placebo and sitagliptin in patients with type 2 diabetes on background metformin monotherapy: a randomised trial. Diabetologia. 2013 Dec;56(12):2582-92. doi: 10.1007/s00125-013-3039-1. Epub 2013 Sep 13. PMID: 24026211; PMCID: PMC3825495.
30. Leiter LA, Yoon KH, Arias P, Langslet G, Xie J, Balis DA, Millington D, Vercruysse F, Canovatchel W, Meininger G. Canagliflozin provides durable glycemic improvements and body weight reduction over 104 weeks versus glimepiride in patients with type 2 diabetes on metformin: a randomized, double-blind, phase 3 study. Diabetes care. 2015 Mar 1;38(3):355-64.
31. Lewin A, DeFronzo RA, Patel S, Liu D, Kaste R, Woerle HJ, Broedl UC. Initial combination of empagliflozin and linagliptin in subjects with type 2 diabetes. Diabetes care. 2015 Mar 1;38(3):394-402.
32. Liao X, Wang X, Li H, Li L, Zhang G, Yang M, Yuan L, Liu H, Yang G, Gao L. Sodium- Glucose Cotransporter 2 (SGLT2) Inhibitor Increases Circulating Zinc- Α2-Glycoprotein Levels in Patients with Type 2 Diabetes. Sci Rep. 2016;6:32887. doi: 10.1038/srep32887.
33. Liu L, Yan H, Xia M, Zhao L, Lv M, Zhao N, Rao S, Yao X, Wu W, Pan B, Bian H, Gao X. Efficacy of exenatide and insulin glargine on nonalcoholic fatty liver disease in patients with type 2 diabetes. Diabetes Metab Res Rev. 2020 Jul;36(5):e3292. doi: 10.1002/dmrr.3292. Epub 2020 Feb 10. PMID: 31955491.
34. Matikainen N, Söderlund S, Björnson E, Pietiläinen K, Hakkarainen A, Lundbom N, Taskinen MR, Borén J. Liraglutide treatment improves postprandial lipid metabolism and cardiometabolic risk factors in humans with adequately controlled type 2 diabetes: A single‐centre randomized controlled study. Diabetes, obesity and metabolism. 2019 Jan;21(1):84-94.
35. Merker L, Häring HU, Christiansen AV, Roux F, Salsali A, Kim G, Meinicke T, Woerle HJ, Broedl UC, EMPA‐REG EXTEND™ MET investigators. Empagliflozin as add‐on to metformin in people with type 2 diabetes. Diabetic Medicine. 2015 Dec;32(12):1555-67.
36. Patel S, Hickman A, Frederich R, Johnson S, Huyck S, Mancuso JP, Gantz I, Terra SG. Safety of ertugliflozin in patients with type 2 diabetes mellitus: pooled analysis of seven phase 3 randomized controlled trials. Diabetes Therapy. 2020 Jun;11(6):1347-67.
37. Pratley RE, Kipnes MS, Fleck PR, Wilson C, Mekki Q. Efficacy and safety of the dipeptidyl peptidase-4 inhibitor alogliptin in patients with type 2 diabetes inadequately controlled by glyburide monotherapy. Obes Metab. 2009;11:167-176.
38. Pratley R, Nauck M, Bailey T, Montanya E, Cuddihy R, Filetti S, Garber A, Thomsen AB, Hartvig H, Davies M, 1860‐LIRA‐DPP‐4 Study Group. One year of liraglutide treatment offers sustained and more effective glycaemic control and weight reduction compared with sitagliptin, both in combination with metformin, in patients with type 2 diabetes: a randomised, parallel‐group, open‐label trial. International journal of clinical practice. 2011 Apr;65(4):397-407.
39. Rosenstock J, Brazg R, Andryuk PJ, Lu K, Stein P, Study S. Efficacy and safety of the dipeptidyl peptidase-4 inhibitor sitagliptin added to ongoing pioglitazone therapy in patients with type 2 diabetes: a 24-week, multicenter, randomized, double-blind, placebo-controlled, parallel-group study. Clinical therapeutics. 2006 Oct 1;28(10):1556-68.
40. Rosenstock J, Kim SW, Baron MA, Camisasca RP, Cressier F, Couturier A, Dejager S. Efficacy and tolerability of initial combination therapy with vildagliptin and pioglitazone compared with component monotherapy in patients with type 2 diabetes. Diabetes, Obesity and Metabolism. 2007 Mar;9(2):175-85.
41. Rosenstock J, Seman LJ, Jelaska A, Hantel S, Pinnetti S, Hach T, Woerle HJ. Efficacy and safety of empagliflozin, a sodium glucose cotransporter 2 (SGLT2) inhibitor, as add‐on to metformin in type 2 diabetes with mild hyperglycaemia. Diabetes, Obesity and Metabolism. 2013 Dec;15(12):1154-60.
42. Rosenstock J, Jelaska A, Zeller C, Kim G, Broedl UC, Woerle HJ, EMPA‐REG BASALTM trial investigators. Impact of empagliflozin added on to basal insulin in type 2 diabetes inadequately controlled on basal insulin: a 78‐week randomized, double‐blind, placebo‐controlled trial. Diabetes, Obesity and Metabolism. 2015 Oct;17(10):936-48.
43. Ross S, Thamer C, Cescutti J, Meinicke T, Woerle HJ, Broedl UC. Efficacy and safety of empagliflozin twice daily versus once daily in patients with type 2 diabetes inadequately controlled on metformin: a 16‐week, randomized, placebo‐controlled trial. Diabetes, Obesity and Metabolism. 2015 Jul;17(7):699-702.
44. Schernthaner G., Schernthaner-Reiter M.H., Schernthaner G.H. EMPA-REG and Other Cardiovascular Outcome Trials of Glucose-lowering Agents: Implications for Future Treatment Strategies in Type 2 Diabetes Mellitus. Clin. Ther. 2016;38:1288–1298. doi: 10.1016/j.clinthera.2016.04.037.
45. Seino Y, Fujita T, Hiroi S, Hirayama M, Kaku K. Efficacy and safety of alogliptin in Japanese patients with type 2 diabetes mellitus: a randomized, double-blind, dose-ranging comparison with placebo, followed by a long-term extension study. Current medical research and opinion. 2011 Sep 1;27(9):1781-92.
46. Seino Y, Sasaki T, Fukatsu A, Ubukata M, Sakai S, Samukawa Y. Efficacy and safety of luseogliflozin as monotherapy in Japanese patients with type 2 diabetes mellitus: a randomized, double-blind, placebo-controlled, phase 3 study. Curr Med Res Opin. 2014;30(7):1245-1255. doi:10.1185/03007995.2014.912983.
47. Shao N, Kuang HY, Hao M, Gao XY, Lin WJ, Zou W. Benefits of exenatide on obesity and non-alcoholic fatty liver disease with elevated liver enzymes in patients with type 2 diabetes. Diabetes Metab Res Rev. 2014 Sep;30(6):521-9. doi: 10.1002/dmrr.2561. PMID: 24823873.
48. Shigiyama F, Kumashiro N, Miyagi M, Ikehara K, Kanda E, Uchino H, Hirose T. Effectiveness of dapagliflozin on vascular endothelial function and glycemic control in patients with early-stage type 2 diabetes mellitus: DEFENCE study. Cardiovascular diabetology. 2017 Dec;16(1):1-2.
49. Shimizu M, Suzuki K, Kato K, Jojima T, Iijima T, Murohisa T, Iijima M, Takekawa H, Usui I, Hiraishi H, Aso Y. Evaluation of the effects of dapagliflozin, a sodiumglucose co-transporter-2 inhibitor, on hepatic steatosis and fibrosis using transient elastography in patients with type 2 diabetes and non-alcoholic fatty liver disease. Diabetes Obes Metab. 2019 Feb;21(2):285-292. doi: 10.1111/dom.13520. Epub 2018 Oct 2. PMID: 30178600.
50. Stenlöf K, Cefalu WT, Kim KA, Alba M, Usiskin K, Tong C, Canovatchel W, Meininger G. Efficacy and safety of canagliflozin monotherapy in subjects with type 2 diabetes mellitus inadequately controlled with diet and exercise. Diabetes, Obesity and Metabolism. 2013 Apr;15(4):372-82.
51. Tang A, Rabasa-Lhoret R, Castel H, Wartelle-Bladou C, Gilbert G, Massicotte-Tisluck K, Chartrand G, Olivié D, Julien AS, de Guise J, Soulez G, Chiasson JL. Effects of Insulin Glargine and Liraglutide Therapy on Liver Fat as Measured by Magnetic Resonance in Patients With Type 2 Diabetes: A Randomized Trial. Diabetes Care. 2015 Jul;38(7):1339-46. doi: 10.2337/dc14-2548. Epub 2015 Mar 26. PMID: 25813773.
52. Wang Y, Xu L, Yuan L, Li D, Zhang Y, Zheng R, Liu C, Feng X, Li Q, Li Q, Ma J. Sodium‐glucose co‐transporter‐2 inhibitors suppress atrial natriuretic peptide secretion in patients with newly diagnosed Type 2 diabetes. Diabetic Medicine. 2016 Dec;33(12):1732-6.
53. Weber MA, Mansfield TA, Alessi F, Iqbal N, Parikh S, Ptaszynska A. Effects of dapagliflozin on blood pressure in hypertensive diabetic patients on renin–angiotensin system blockade. Blood pressure. 2016 Mar 3;25(2):93-103.
54. Wilding JP, Woo V, Rohwedder K, Sugg J, Parikh S, Dapagliflozin 006 Study Group. Dapagliflozin in patients with type 2 diabetes receiving high doses of insulin: efficacy and safety over 2 years. Diabetes, Obesity and Metabolism. 2014 Feb;16(2):124-36.
55. Yale JF, Bakris G, Cariou B, Yue D, David‐Neto E, Xi L, Figueroa K, Wajs E, Usiskin K, Meininger G. Efficacy and safety of canagliflozin in subjects with type 2 diabetes and chronic kidney disease. Diabetes, Obesity and Metabolism. 2013 May;15(5):463-73.
56. Yan J, Yao B, Kuang H, Yang X, Huang Q, Hong T, Li Y, Dou J, Yang W, Qin G, Yuan H, Xiao X, Luo S, Shan Z, Deng H, Tan Y, Xu F, Xu W, Zeng L, Kang Z, Weng J. Liraglutide, Sitagliptin, and Insulin Glargine Added to Metformin: The Effect on Body Weight and Intrahepatic Lipid in Patients With Type 2 Diabetes Mellitus and Nonalcoholic Fatty Liver Disease. Hepatology. 2019 Jun;69(6):2414-2426. doi: 10.1002/hep.30320. Epub 2019 Feb 22. PMID: 30341767; PMCID: PMC6594101.
57. Zhang LY, Qu XN, Sun ZY, Zhang Y. Effect of liraglutide therapy on serum fetuin A in patients with type 2 diabetes and non-alcoholic fatty liver disease. Clinics Res Hepatol Gastroenterol (2020) 44(5):674–80. 10.1016/j.clinre.2020.01.007
